# 

**Published:** 1882-01

**Authors:** 


					﻿Whole Number,
CCXXXIV,
JANUARY, 1882.
Number 6
Volume XXI.
THE
BUFFALO
Medical and Surgical Journal.
EDITORS :
THOS. I.OTHROP, M. D.,
P. W. VAN PEYMA, M.
A. R. DAVIDSON, M.
EUCIEIN HOWE, M.
HERMAN MYNTER, M. D.

BUFFALO :
Published by the Medical Journal Association.
Read the Notice of this Journal among the Advertisements.
Entered at the Post-Office at Buffalo, N. Y., as Second-Class Mail Matter.
				

